# Maximum reduction of energy losses in multicore MgB_2_ wires by metastructured soft-ferromagnetic coatings

**DOI:** 10.1038/s41598-022-10728-5

**Published:** 2022-04-29

**Authors:** M. Kapolka, H. S. Ruiz

**Affiliations:** School of Engineering and Space Park Leicester, University of Leicester, University Rd, Leicester, LE1 7RH UK

**Keywords:** Engineering, Electrical and electronic engineering, Superconducting properties and materials, Materials for devices

## Abstract

When compared with rare-earth coated conductors, magnesium diboride superconducting cables are known to show significant advantages by cost and easy production. However, the inherent difficulty for achieving a significant reduction of their magnetization losses in multifilamentary wires, without degrading the high critical current density that is so characteristic of the monowire, is considered as one of the major drawbacks for their practical use in high power density applications. Being this one of the major markets for superconducting cables, from fundamental principles and computational optimization techniques, in this paper we demonstrate how the embedding of the superconducting filaments into soft-ferromagnetic metastructures can render to their full magnetic decoupling, and therefore, to the maximum reduction of the energy losses that can be achieved without deteriorate the critical current density of the cable. The designed multifilamentary metastructure is made of NbTi coated MgB_2_ superconducting filaments in a Cu-matrix, serving as a reference for validating our model with actual experimental measurements in monowires and multifilamentary wires. The novelty in our computationally aided multifilamentary wires, is that each one of the filaments is embedded within a thin metastructure made of a soft-ferromagnetic layer and a resistive layer. We have found that for soft-ferromagnetic layers with magnetic permeabilities in the range of $\mu _{r}=$ 20–100, nearly a full magnetic decoupling between the superconducting filaments can be achieved, leading to efficiencies higher than $92\%$, and an overall reduction of the AC-losses (including eddy currents at the Cu-matrix) higher than $50\%$.

## Introduction

Despite the enormous progress on the commercialization of high temperature superconducting (SC) wires for high magnetic field and power systems applications^1–5^, still the so-called low temperature superconductors such as NbTi, Nb$_{3}$Sn, and MgB$_{2}$ with critical temperatures, $T_{c}$, ranging between $\sim$10 K to $\sim$39 K, dominate the industrial market^6–8^. If compared with the manufacturing of coated-cuprates conductors, their success is due to their low-cost per unit length, their ease fabrication, and the relative abundance of their base compounds. In this regard, round MgB$_{2}$ chemically doped wires are of particular importance, not only due to their superior $T_{c}$ and upper critical field, $H_{c2}$, but because the powder-in-tube process allows an easy manufacturing of multicore (a.k.a. multifilamentary) wires^9,10^, as precursors of high-power AC/DC applications such as, power transmission cables^11–13^, rotary machines^14–17^, energy-storage devices^18^, MRI cryogen-free magnets^19–21^, fusion reactors^22^, and the large scale commercialization of high-current SC links for CERN^23,24^.

However, the need to operate at temperatures well below sub-cooled liquid nitrogen (65–77 K)^25^, and the high costs associated with the handling of their energy losses under AC conditions, rise significant issues onto the use of MgB$_{2}$ wires for power transformers, fault current limiters, and HV-AC cables, capable to function with commercial cryocoolers at sub-cooled LH2-temperatures (15–33 K). Therefore, to enable MgB$_{2}$ wires tap into the market of AC applications, it results imperative to find meaningful ways to reduce their AC-losses without a detriment of its critical current density, $J_{c}$. Thus, in this paper we present a novel optimization method applied to realistic designs for multifilamentary MgB$_{2}$ wires, which aided by the magnetic diverter principle of soft ferromagnetic coatings over superconducting wires^26–37^, can render to the maximum reduction of energy losses than can be achieved by first-physical principles. In this sense, we provide a detailed route for the adequate selection of materials, physical, and engineering dimensions, which in an optimal combination can lead to the fabrication of cost-effective metastructured multifilamentary MgB$_{2}$ wires for high power density AC applications, that are nowadays accessible only by the use of much more expensive rare-earth coated conductors.

## Possible alternatives for the reduction of energy losses in a MgB$_{2}$ wire

Finding ways for reducing the AC-losses in a SC wire while maintaining its $J_{c}$ is not trivial, as these are fundamentally constrained by the material properties of the composite, and the robustness of its magnetic flux pinning. Thence, microscopical or macroscopical approaches can be taken, with the first commonly focused on the crystallographic or microstructural properties of the SC compound, despite neither their role on the pairing mechanism leading to the SC phenomenon^38–40^, nor their influence on the vortex pinning dynamics^41–44^ are fully understood.

On the other hand, multiple approaches based upon the celebrated Bean’s model have arisen^45,46^, whose success lies on the averaging of all flux pinning forces within a simple magnetostatic scenario. This allows to accurately determine the SC energy losses, with the SC electrical-conductivity known either by intrinsic mechanisms as the one described by the critical state model (CSM)^47,48^, or by extrinsic measurements of the highly non-linear relationship between the electric field, $\mathbf{E}$, and the current density, $\mathbf{J}$, also known as $\mathbf{E} -\mathbf{J}$ power law^49,50^. However, it is under the CSM on which it is possible to determine the actual (analytical) physical minimum for the hysteretic AC losses per unit time and volume, *L*, at least in the case of cylindrical SC wires subjected to external current or magnetic field excitations of frequency $\omega$^51,52^. This can be calculated then by integration of the local density of power dissipation $(\mathbf{E} \cdot \mathbf{J} )$ over the SC volume $(\Phi )$ as,

1 $$\begin{aligned} L = \omega \oint _{f.c} dt \int _{\Phi } \mathbf{E} \cdot \mathbf{J} \, d\Phi \, , \end{aligned}$$

where *f*.*c*. denotes a full cycle of the time-varying electromagnetic excitation.

This fact sets a benchmark for which any design of rounded SC wires must be tested^53–56^, and from which possible pathways for the reduction of its energy losses can be assessed. Thus, in the CSM only *J* is constrained by $J \le J_{c}$, allowing to calculate the minimum energy losses per unit volume of a cylindrical wire of SC cross section area, $A_{SC}=\pi R_{SC}^{2}$, by^57^

2 $$\begin{aligned} L \equiv \dfrac{\mu _{0} I_{c}^{2}}{4\pi ^{2}R_{SC}^{2}}\left[ \left( i_{tr}-\dfrac{i_{tr}^{2}}{2}\right) +\left( 1-i_{tr}\right) \ln \left( 1-i_{tr}\right) \right] , \end{aligned}$$

with $i_{tr}$ the ratio between the amplitude of the transport current, $I_{tr}$, and the critical current, $I_{c}=J_{c}A_{SC}$.

**Table 1:** Material properties for the MgB$_{2}$ wires considered (see insets at Figs. 1, 2).

Parameter*	Monocore	Multicore	Metastructure
MgB$_{2}$ Filaments (SC)	1	6	6
Radius, $R_{SC}$ [µm]	275	65	65
Temperature, *T* [K]	26.4	4.2	20
$J_{c}$ [GA/m$^2$]	0.8	5.87	1–2^58^
$I_{c}$ [A]	192	800	80–160
Barrier (B)	Nb	Nb	Nb(Ti)
$\delta _{B}$ [µm]	67	20	10–30
$\rho _{B}$ [$\mu \Omega \cdot$cm]	0.61	–$^{\dag }$	$\rho _{B}$ $^{\ddagger }$
Sheath (S)	Cu	Glidcop	Glidcop
$\delta _{S}$ [µm]	58	125	80
$\rho _{S}$ [$\mu \Omega \cdot$cm]	0.01	16.1	16.1 $^{\S }$
Multicore matrix (M)	–	Cu	Cu
Radius, $R_{M}$ [µm]	–	290	370
$\rho _{M}$ [$\mu \Omega \cdot$cm]	–	0.564	0.564$^{\S }$
Metastructure	–	–	SFM\RE
$\delta _{SFM}$ [µm]	–	–	10–30
$\delta _{RE}$ [µm]	–	–	10–30
$\rho _{SFM/RE}$ [$\mu \Omega \cdot$cm]	–	–	$\gtrsim$ 10$^{\P }$
Wire radius [µm]	400	415	450

*Material properties of the Monocore and Multicore wires from^59,60^, respectively . $\delta _{x}$ and $\rho _{x}$ define the thickness and electrical resistivity of the $x-$material.

$\dag$At $T=4.2$ K the Nb barrier becomes SC following the same power law for the MgB$_{2}$^61^, i.e., $E(J)=E_{c} (|J|/J_{c})^{n}$, with $E_{c}=1~\mu$V/cm, $J_{c}=I_{c}/\pi (R_{SC}+B_{thick})^{2}$, and n=116.

$$^{\ddagger }$$$$\rho _{B}(T=20$$K$$)=0.5\mu \Omega \cdot $$cm for the Nb barrier^59^ and 1.91 $$\mu \Omega \cdot $$cm for NbTi^58^.

$\S$Electrical resistivity of Cu-based metal composites is not expected to change below $\sim 20$ K^62,63^.

$^{\P }$ Calculations have been made for 10–10,000 $\mu \Omega \cdot$cm, with no apparent change on *L* within a tolerance of 1E−9 J/m.

**Figure 1:**
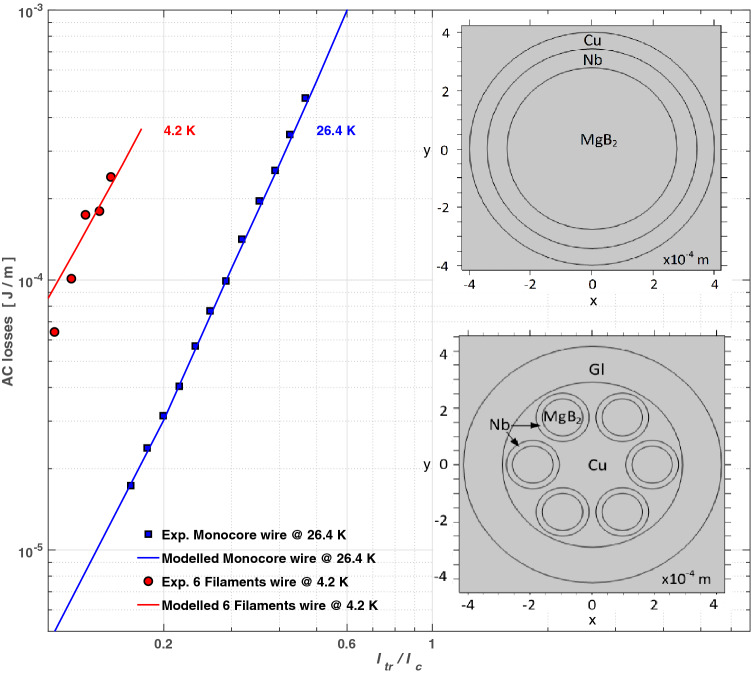
Calculated AC-losses for the MgB$_{2}$ monocore and multicore wires shown at the right-pane inset. Solid symbols correspond to the experimental measurements at 26.4 K for the monocore wire^59^, and 4.2 K for the six filaments multifilamentary (multicore) wire^60^.

Then, for determining the total energy losses in realistic MgB$_{2}$ wires, we have to include the losses from the other materials involved. Thus, we considered three different designs named a monocore, a multicore, and a metastructured wire, with their material properties given in Table 1. The monocore and the multicore designs have been used as benchmark (right-insets at Fig. 1), as the experimental measurement of their AC-losses is known^59,60^. Ergo, for reproducing these experiments we have used the so-called H-formulation^64,65^, with our results shown as colour solid lines in Fig. 1.

Now, there are multiple pathways to be considered when optimizing SC multifilamentary wires. On the one hand, there are extrinsic techniques like chemical doping^66–68^ or irradiation treatments^69–71^, which both can enhance the $J_{c}$ by improving the flux pinning mechanism, but do not change that the AC-losses will still be dominated by the contributions of the SC filaments and its metallic matrix. On the other hand, extrinsic methods such as the twisting of the filaments can lower the AC-losses, but at the expense of an undesired reduction of $J_{c}$^58^. Twisting the filaments exploits two physical phenomena not yet well understood, firstly, the angular dependence of the MgB$_{2}$ pinning properties^72–74^ and, secondly, the possible occurrence of flux cutting effects by the non-perpendicularity between the magnetic field and $I_{tr}$^75–78^. The combination of these two phenomena can lead to an “apparent” magnetic decoupling of the SC filaments, apparent in the sense that it can lead to an equivalent reduction of the AC-losses as if no mutual induction would exist. However, there is no general rule in regards to the twisting of MgB$_{2}$ filaments, as technically speaking, a true magnetic decoupling cannot be achieved unless the mutually induced field between filaments is diverted with the aid of a ferromagnetic material. Hence the advantage of our approach, where the filaments can get truly decoupled by embedding them within a soft ferromagnetic (SFM) sheath, whilst an electrically resistive (RE) layer reduces the losses at its metallic matrix.

The losses by the metallic matrix are due to the strength and change of the magnetic field created by the AC in the MgB$_{2}$ filaments, whilst the filament losses are due to the $I_{tr}$ and the self- and mutually-induced magnetization currents appearing in type-II superconductors^53–56^. Same happens if other materials with higher thermal conductivity and tensile properties are used for the barrier and outer sheath, such as Nb-Ti, Cu-Nb, or Monel alloys^79,80^, as they have an almost negligible impact on the total losses of the SC wire^58–60,81,82^. Therefore, the key for achieving a true reduction of the AC-losses lies on finding how to simultaneously lower the magnetically induced currents in the SC filaments, whilst reducing the eddy currents at its metallic matrix. This should be achieved by embedding the SC filaments into ferromagnetic shields as theorized by Majoros, Glowacki and Campbell (MGC) in [^83^], although contrary to their predictions, a counterintuitive increment on the energy losses has been reported^84–90^.

## Optimal metastructure for maximal reduction of AC losses

**Figure 2:**
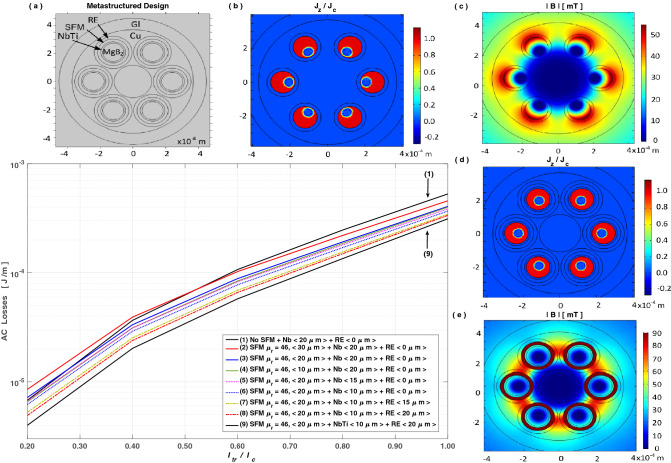
AC losses for multiple (9) MgB$_{2}$ wires with the thicknesses of the barrier, SFM, and RE layers labelled in quotation marks. (**a**) shows the metastructured SC wire concept, with the profiles of (**b**-**d**) $J_{z}/J_{c}$ and (**c**-**e**) $|\mathbf{B} |$ displayed for (1) a conventional-wire, and (9) the optimized metastructured-wire, at the peak transport current $I_{tr} = 0.8 I_{c}$, and with $J_{c}=1$ GA/m$^2$ as reference.

Based on our recent findings on the actual magnetostatic coupling between type-II SC wires and SFM materials^37^, in this section we explain not only the physical reasons behind the reported increment on the AC losses above mentioned, but also provide a solution pathway for achieving the maximum reduction of energy losses feasible in multifilamentary rounded MgB$_{2}$ wires. For doing so, in Fig. 2 we present our results for the AC-losses of the considered 6 filaments MgB$_{2}$ wire under an applied current $I_{a}=I_{tr} \sin (\omega t)$ at low frequency, e.g., (50 Hz), but where each filament is metastructured with an additional SFM layer within a “resistive” (RE) sheath, whose electrical resistivity is at least a couple of orders greater than the one of the Multicore Matrix (see Table 1). In this sense, if the multicore matrix is made of Cu, with an estimated electrical resistivity of 0.564 $\mu \Omega \cdot$cm at 20 K (see Table 1), the RE layer could be made of alloys such as Hastelloy, Inconel, Udimet, or graded AISI austenitic steels^91^.

Furthermore, it is worth reminding that in conventional power systems encountering excitation frequencies greater than 50–60 Hz is uncommon, but more importantly is to remind that at least within the so-called quasi-steady low frequency regime (below radio frequencies $\sim$ 20 kHz), the superconductor and the ferromagnetic material can be modelled within the framework of the critical state theory with relatively no differences in he hysteretic losses^37,92–94^. Therefore, not only the standard H-formulation can still be used for determining the distribution of current density inside the superconducting filaments, but the eddy currents to be produced by the SFM and RE layers are not expected to overshadow the hysteretic losses produced by the superconductor, as long as this study is considered within the low frequency regime.

The natural trend for the optimization process can be seen from the right ordinate of the main plot in Fig. 2, i.e., for $i_{tr}=I_{tr}/I_{c}=1$, where the curves are top to bottom numbered. Starting with the wire with highest losses (1), this curve corresponds to the case where no SFM\RE metastructure is added, therefore consistent with the results and design shown in Fig. 1. Then, the following curves, (2)–(4), correspond to metastructured MgB$_{2}$ wires with only the SFM filamentary sheaths added. Their purpose is to illustrate the effect of the SFM thickness on the total AC-losses of the MgB$_{2}$ wire, when a SFM layer of relative magnetic permeability $\mu _{r}$ coats each of the Nb(Ti)/MgB$_{2}$ filaments. The trends found with the Nb or the NbTi barrier cases are the same, reason why we call it categorically as Nb(Ti). Also, the selection of this $\mu _{r}$ is not arbitrary, as a $\mu _{r}>100$ has serious repercussions on the total AC-losses of the MgB$_{2}$ wire^37^. These aspects will be revised later, but for now it suffices to mention that a SFM with $\mu _{r}=46$ has been chosen, as it has been previously used for fabricating MgB$_{2}$ monocore wires^95–98^. Moreover, it has been assumed as an ideal SFM with a very small coercive force and a sufficiently high saturation field, such that a constant relative magnetic permeability could be assumed. In this sense, although our results stablish a sound benchmark for enabling the fabrication of MgB$_{2}$ metastructures with minimal AC losses (near the analytical limit), the actual choice and thickness of the SFM must be taken by having into consideration its saturation field, which should be higher than the maximum filed produced by the SC wire.

Hence, it is to be noticed that for a $30\mu$m SFM layer, i.e., 1.5 times thicker than the Nb(Ti) barrier (Fig. 2, curve (2)), the total AC-losses at moderate to low transport currents, $I_{tr}<0.5I_{c}$, are actually greater than the losses for the conventional multicore wire (curve (1)), with lower losses only seen as $I_{tr}$ approaches $I_{c}$. However, by reducing the SFM-thickness to equals the one of the barrier (curve (3)), a sooner and stronger reduction of the AC-losses can be seen, with a nearly negligible change for $i_{tr}\le 0.3$. Then, by further reducing the SFM-thickness to e.g., 10 µm (curve (4)), i.e., half the barrier-thickness, nearly no further reduction on the AC-losses can be observed without the RE-layer. Even if we include the RE-layer (curves 7–9), no further reduction on the AC-losses for $\delta _{SFM}<15\mu$m has been found. Therefore, not only choosing a too thick SFM layer could be detrimental, but choosing a thinner SFM layer than the barrier do not implies a continuous reduction on the AC-losses. Thus, our optimization process leads to conclude that an ideal thickness for the SFM sheath would be around the same thickness of the Nb(Ti) barrier, i.e., $\sim$ 20 µm in our case, with a saturation field greater than 60 mT (Fig. 2c). Nevertheless, we must assess a relative optimization pathway for the barrier’s thickness.

The thickness of the Nb(Ti) barrier has been chosen to be $\pm 10~\upmu$m the real thickness used for the multicore MgB$_{2}$ wire in Fig 1 ($20~\upmu$m), resulting in a negligible impact on the AC-losses for $\delta _{Nb(Ti)}\ge 15~\mu$m (Fig. 2, curves (4) & (5)), and only a slight reduction when it is reduced to $\delta _{Nb(Ti)}=10~\mu$m (curve (6)). Therefore, although a significant pathway for reducing the AC-losses at high transport currents in multifilamentary MgB$_{2}$ wires has been found, yet with an optimal $20~\upmu$m SFM layer, the AC-losses reduction is still insignificant at low to moderate $I_{tr}$. Thus, although we have managed to get a nearly full decoupling of the *J*-profiles inside the SC filaments (see subplots (b) and (d) at Fig. 2), leading to an actual reduction of the AC-losses for the entire wire, still the advantage of including the SFM sheaths is overshadowed by the eddy currents at the multicore matrix. In this sense, it results vital to understand the physics that leads the reduction of the AC-losses when the SFM is chosen with $\mu _{r}<100$.

This is no other than the impact of the magnetostatic coupling between the SC and the SFM sheath shown by Fareed and Ruiz in monocore-wires^37^, whom have demonstrated that no matter the SFM used nor its dimensions, for $\mu _{r}\gtrsim 100$, the physical coupling between the SC and the SFM leads to a large increment on the electric field outside the SC\SFM metastructure^35^, but not to a further increment in the density of power losses $\mathbf{E} \cdot \mathbf{J}$ inside the SC. Moreover, this coupling gets dominated by the factor,

3 $$\begin{aligned} {\bar{R}}_{\mu 1}=\dfrac{R_{SFM}^{2n}-R_{SC}^{2n}}{\mu _{(-)}^{2} R_{SC}^{2n} - \mu _{(+)}^{2} R_{SFM}^{2n}} \, , \end{aligned}$$

where $\mu _{(\pm )}=\mu _{r}\pm 1$, being $R_{SFM}$ the SFM-sheath outer radius and $R_{SC}$ its inner radius (outer radius of the Nb(Ti)/MgB$_{2}$ filament in our case). Thus, following the analytical methods in [^37^], we have derived the additional contribution for the magnetic vector potential caused by the coupling between a SC\SFM sheathed filament and a line of current inside another SC\SFM filament, i.e.,

4 $$\begin{aligned} \mathbf{A} _{c,j}(\cdot )= \dfrac{\mu _{0}\mu _{(-)}^{2}}{2\pi }{} \mathbf{J} _{i} \left[ \sum _{n=1}^{\infty } \dfrac{{\bar{R}}_{\mu 1}}{n} \left( \dfrac{r_{j}}{r_{i}}\right) ^{n} \cos (n\phi _{j}) \right] \, . \end{aligned}$$

Here, $\mathbf{J} _{i}$ is the current density at an element located at the *i*th filament ($i \ne j$), with $r_{i}$ the distance between the center of the targeted *j*th filament and, the element of current inside the magnetically coupled *i*th filament. Then, the coordinates ($r_{j},\phi _{j}$) refer to the polar expansion components of the current elements inside the targeted *j*th SFM/SC filament. In consequence, the additional contribution on the magnetic vector potential, and therefore on its derived quantities, $\mathbf{B} =\nabla \times \mathbf{A}$, $\mathbf{E} =-d\mathbf{A} /dt$, and consequently, $L\propto \mathbf{E} \cdot \mathbf{J}$, are also governed by the factor, ${\bar{R}}_{\mu 1}$. Therefore, this reveals that for SFM sheaths with $\mu _{r}>100$, no further enhancement of its “*shielding*” properties is to be expected for any $R_{SFM}/R_{SC}$, as for these magnetic permeabilities, ${\bar{R}}_{\mu 1}$ tends to zero [^37^].

However, in order to balance the sudden increment in the magnetic flux density caused by the SFM, it is already known that near the outer surface of a SC\SFM metastructure, the strength of **B** and **E** change rapidly from a much higher value ($\propto \mu _{r}$) than the one expected for a bare SC filament^37^. Therefore, an increment in the eddy currents at the metallic matrix is to be expected, which is likely to increase the overall losses of the MgB$_{2}$ wire either by an inadequate choice of the SFM $\mu _{r}$, or by the lack of a RE layer. Thus, in Fig. 2, curves (6)–(8), we show the effect of adding a RE sheath to the metastructured filaments, for which a significant reduction of the AC-losses has been achieved even at low $i_{tr}$ for $\delta _{RE}>10~\mu$m. Likewise, this concept is proved by increasing the barrier’s electrical resistivity, e.g., by changing it from Nb to NbTi (Table 1), with which a further AC-losses reduction can be conceived (curve (9)).

Remarkably, when considering other important engineering parameters such as the engineering current density for multicore or metastructured wires, it is to be noted that the designing of metastructured wires can truly result in promising candidates for the next generation of MgB$_{2}$ based technologies. For instance, if the same critical current density of the superconducting material can be maintained with either of the designs, for instance a $J_{c} = 1$ GA/m$^{2}$, leading to a total $I_{c}$ of 79.6 A per wire (when considering six MgB$_{2}$ filaments each of 65 µm radius), by increasing the overall radius of the wire from, let’s say, 415 µm to 450 µm when including the SFM\RE metastructure, the engineering current density of the wire is decreased by only a 15%. Then, this can be even further reduced to 0%, by simply reducing the thickness of the wire outer coating, i.e., the 80 µm of Glidcop considered in our case, to just 45 µm, what leaves the estimation of the AC losses unaffected. Nevertheless, complementary studies on the tensile properties of the wire should be conducted to determine the optimal and minimum thickness of the outer layer, indistinctly of whether it is the simplified multicore wire or the metastructured wire.

## Concluding remarks

**Figure 3:**
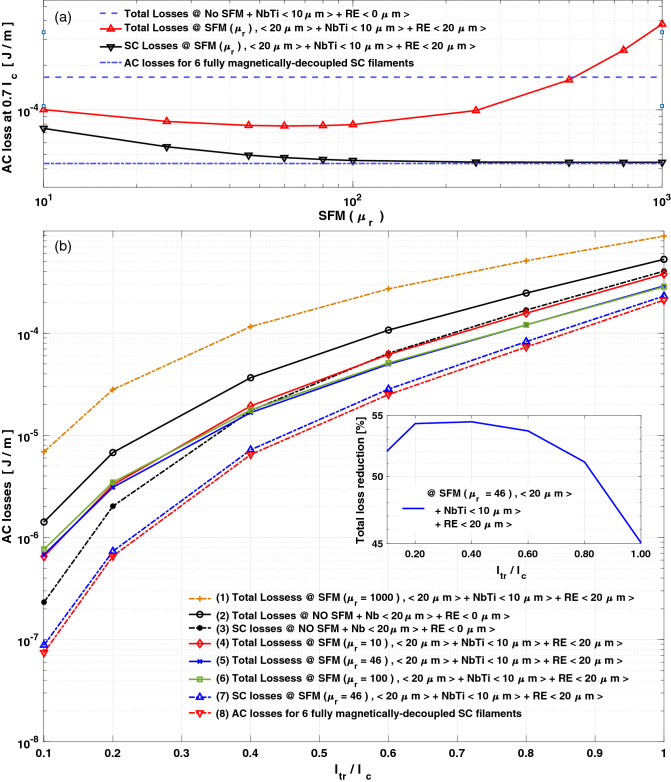
AC-losses for the 6-filaments metastructured wire as a function of (**a**) the SFM $\mu _{r}$ at $I_{tr}=0.7 I_{c}$, and (**b**) the $I_{tr}$ condition with different SFM $\mu _{r}$ options (curves 1–6), leading to the optimal design (5), where a nearly perfect magnetic decoupling between SC filaments is shown (7–8). The inset shows the total AC-loss reduction for the optimal design.

By further analysing the impact of the SFM sheath on the AC-losses of SC\SFM\RE metastructured wires (Fig. 3a), starting from a bare SC $(\mu _{r}=1)$, up to a high magnetic permeability $(\mu _{r}=1000)$, i.e., including SFMs such as Ni, NiZn, MnZn, Si, C, and Co ferrites^99–101^, our calculations confirm that for SFM sheaths with $\mu _{r}>100$, no further reduction of the SC losses can be achieved, but an increment due to eddy currents at the Cu matrix. Thus, Fig. 3b summarizes our main findings, showing how the AC-losses for a SC\SFM\RE wire strongly depends on the SFM selection.

For instance, it explains why when the SFM $\mu _{r}$ is too high, e.g., $\mu _{r}=1000$ (curve-(1)), a significant increment on the overall AC-losses is to be observed^84–90^. Moreover, with a SFM sheath of $\mu _{r}\le 100$ and the RE layer, we have proven that the maximum AC-loss reduction (a factor of $\sim 2$) predicted by MGC^83^, can be realized. This is seen by comparing the full (curve-(2)) and the SC (curve-(3)) AC-losses for a conventional MgB$_{2}$ multifilamentary-wire without the SFM\RE metastructure, with the total losses of the SC\SFM\RE metastructured-wire with a 20 µm SFM sheath of $\mu _{r}=10$ (curve-(4)), $\mu _{r}=46$ (curve-(5)), or $\mu _{r}=100$ (curve-(6)), all embedded within a 20 µm RE layer. Thus, although for low to moderate currents, i.e., for $I_{tr}\le 0.4I_{c}$, there is a clear reduction of the AC-losses in the order predicted by MGC (see the inset), the wire losses at this regime are mainly dominated by the eddy currents at the Cu-matrix, somehow hiding the achievement of a nearly full magnetic decoupling between the SC filaments. However, the decoupling between the SC filaments becomes clearer when practical values of the current $(I_{tr} > 0.4 I_{c})$ are assumed. For instance, by choosing a SFM with $\mu _{r}=10$, this metastructured-wire leads to at least the same losses predicted for only the SC filaments in a conventional multifilamentary-wire. In other words, although we still have eddy current losses at the non-SC materials, the total losses including the SC filaments results equivalent to discounting the losses created by the non-SC materials in conventional multifilamentary wires. Moreover, by increasing the $\mu _{r}$ of the SFM layer to $\mu _{r}=46$ and then, up to the predicted limit of $\mu _{r}=100$, it is observed that the total AC-losses of the metastructured-wire result lower than the sole SC losses for the conventional multifilamentary-wires. Therefore, reminding that the SC losses come from the concomitant action between the transport current and the magnetic inductance in the SC filaments^56^, and neither the transport current losses nor the self-field losses can be reduced as they are bounded by the CSM^47^, then this can only means the magnetic decoupling between the SC filaments. This is shown by comparing the two bottom curves at Fig. 3b, where the SC losses for the metastructured-wire with SFM $\mu _{r}=46$ (curve (7)), is contrasted with the analytical solution (Eq. 2) for six fully decoupled SC filaments. Thence, this letter demonstrates that a nearly full decoupling of the SC filaments can be achieved by the right choice of materials within a SC\SFM\RE metastructure, finding a pathway for the maximum reduction of AC-losses envisaged for MgB$_{2}$ wires.

## Data Availability

The data that supports the findings of this study are available within the article and from the corresponding author upon reasonable request.
